# Anacardic Acid-Modified Fe_3_O_4_ Nanoparticles: Synthesis, Characterization, and Thermal and Tribological Performance in Biolubricants

**DOI:** 10.1021/acsomega.6c03439

**Published:** 2026-07-08

**Authors:** David Alves de Assis, Denise Ramos Moreira, Alexandre Carreira da Cruz Sousa, Antonia Flávia Justino Uchoa, Felipe Bohn, Walney Silva Araujo, Nágila Maria Pontes Silva Ricardo

**Affiliations:** † Laboratory of Polymers and Materials Innovation, Department of Organic and Inorganic Chemistry, 28121 Federal University of Ceará, Fortaleza, Ceará 60440-900, Brazil; ‡ Federal Institute of Education, Science and Technology of Ceará, Quixadá Campus, Quixadá, Ceará 63902-580, Brazil; § Department of Transportation, Federal University of Ceará, Fortaleza, Ceará 60020-181 Brazil; ∥ Department of Physics, 28123 Federal University of Rio Grande do Norte, Natal, Rio Grande do Norte 59078-900, Brazil; ⊥ Department of Metallurgical and Materials Engineering, 28121 Federal University of Ceará, Fortaleza, Ceará 60440-554, Brazil

## Abstract

Magnetic iron oxide nanoparticles (Fe_3_O_4_) functionalized with anacardic acid (AAc), producing Fe_3_O_4_@AAc nanoparticles, were synthesized via chemical coprecipitation to improve the performance of biolubricants. The nanoparticles were characterized in terms of their structure, morphology, thermal properties, and magnetism. They exhibited a spherical morphology with an average diameter of 14.7 ± 4.2 nm, demonstrated good thermal stability, and contained approximately 12.4 wt % surface-bound AAc. Superparamagnetic behavior was preserved for the functionalized nanoparticles. To evaluate their effectiveness as performance-enhancing additives, Fe_3_O_4_@AAc nanoparticles were incorporated into a trimethylolpropane trioleate (TMPTO)-based biolubricant at concentrations of 0.05 and 0.15 wt %. Adding 0.05 wt % improved thermal resistance, and adding 0.15 wt % enhanced rheological stability, reducing both the coefficient of friction (COF) and wear. The nanoparticles lowered the activation energy for viscous flow and maintained Newtonian behavior up to 80 °C at 0.15 wt %. Tribological tests indicated that the 0.15 wt % formulation exhibited the lowest COF (0.036), a reduced wear scar diameter (WSD) (0.342 mm), and formation of a more stable protective tribofilm compared to pure biolubricant and reference mineral oil. These results confirm that anacardic acid functionalization promotes effective nanoparticle dispersion, with the magnetic nanoparticles acting as efficient additives that improve the thermal and tribological performance of the biolubricant.

## Graphical abstract

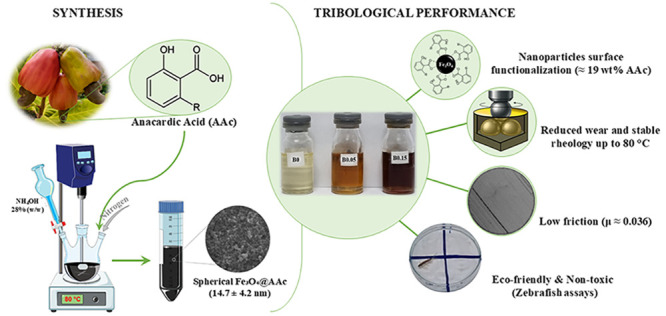

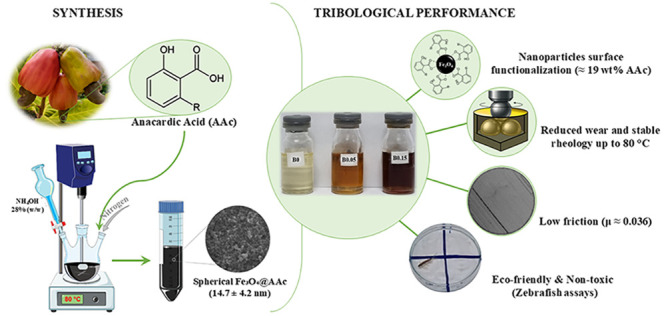

## Introduction

Lubricants play a fundamental role in reducing friction, minimizing wear, and ensuring the efficient operation of mechanical systems.^1^ However, most commercial lubricants are derived from petroleum, a nonrenewable resource associated with environmental concerns, such as low biodegradability and ecotoxicity.^2^ These limitations have intensified the global demand for sustainable alternatives, driving the development and application of biolubricants.

Derived from renewable sources such as vegetable oils or obtained via synthesis (synthetic esters), these materials offer key advantages: high biodegradability, low toxicity, and a chemical structure rich in functional groups, which confers excellent lubricating performance.^3^ However, despite these benefits, their practical application is still restricted by disadvantages, including susceptibility to oxidation, limited use in high-performance or high-temperature environments, and inadequate cold-flow properties.^4^

Due to the limitations of base oils when used as lubricants, additives are used to modify their properties and improve the performance of the final lubricant. Among these strategies, incorporating nanoparticles has emerged as a promising way to improve the physicochemical properties of biolubricants. Nanoparticles can reduce friction, increase wear resistance, promote the formation of protective tribofilms, and improve thermal and oxidative stability.^4–6^ However, their effectiveness depends heavily on their ability to remain well-dispersed in the lubricant matrix. The low compatibility between inorganic nanoparticles and hydrophobic biolubricant matrices often results in agglomeration, sedimentation, and reduced performance. Thus, the surface modification of nanoparticles through functionalization is essential to improve dispersion stability and optimize lubrication efficiency.^7,8^

Among the nanomaterials of interest, magnetite nanoparticles (Fe_3_O_4_) stand out due to properties such as ferromagnetism, chemical stability, adjustable size, and low production cost, which make them promising candidates as functional additives in lubricants.^5,6,9,10^ However, the inherent low affinity of nonfunctionalized iron oxide nanoparticles for hydrophobic organic matrices hinders their dispersion and limits their tribological performance.^11^ In light of this, surface functionalization with organic molecules emerges as an essential strategy to increase compatibility and create more efficient nanostructured additives.

In this context, anacardic acid (AAc) (Figure S1) has emerged as a notably promising biobased molecule for the functionalization of magnetic nanoparticles. AAc, a natural phenolic compound derived from the liquid of the cashew nut shell liquid (CNSL), is a mixture of homologous compounds that share the same salicylic acid core, differing in the degree of unsaturation of the C15 alkyl side chain. This feature makes it particularly attractive for nanoparticle functionalization due to its amphiphilic structure.^12^ The molecule has a phenolic group capable of strongly bonding to metal oxide surfaces and a long hydrophobic aliphatic chain that increases compatibility with organic media. These characteristics provide a dual function: stabilizing the nanoparticles and promoting their dispersion in biolubricants.^13,14^ However, despite this potential, the application of nanomaterials functionalized with anacardic acid as tribological additives in sustainable oil-based matrices remains underexplored in the literature.

This work aimed to extract and isolate anacardic acid from cashew nut shell liquid, synthesize and characterize magnetic iron oxide functionalized with anacardic acid (Fe_3_O_4_@AAc) nanoparticles, and evaluate their performance as functional additives in a TMPTO-based biolubricant (Figure S1). The study investigates the thermal behavior, rheological stability, magnetic and tribological properties of formulations containing different nanoparticle concentrations, as well as their acute toxicity and neurobehavioral effects in adult zebrafish. By integrating physicochemical characterization with biological assessment, this research provides a comprehensive analysis of the potential of Fe_3_O_4_@AAc nanoparticles as a sustainable, high-performance additive for advanced biolubricant formulations.

## Materials and Methodology

### Materials

Iron­(II) chloride tetrahydrate ≥99%, iron­(III) chloride hexahydrate ≥99%, ethyl acetate anhydrous ≥99.8%, calcium hydroxide ≥95.0%, ethanol P.A., and anhydrous sodium sulfate ≥99% were obtained from Sigma-Aldrich (USA). Ammonium hydroxide 30–50% and hydrochloric acid P.A. were purchased from Synth (Brazil). Ultrapure water (Milli-Q Advantage A-10 system, Millipore) was used in this work. Fresh cashew nuts were obtained from Caucaia, CE, Brazil. TMPTO was synthesized and donated by our research group (Polymer and Materials Innovation Laboratory) and used as received. Adult wild-type *Danio rerio* specimens were obtained from a commercial supplier, Agroquímica: Comércio de Produtos Veterinários LTDA (Ceará, Brazil). All chemicals used were analytical grade and were used as received.

### Methodology

#### Extraction Of Cashew Nut Shell Liquid (CNSL) and Isolation of Anacardic Acid (AAc)

CNSL was extracted from cashew nut shells using ethyl acetate following a modified procedure based on Khadrawy et al.^15^ The extract was concentrated under reduced pressure to afford CNSL (20.3% w/w yield). Anacardic acid (AAc) was subsequently isolated from CNSL according to a modified method described by Collymore et al.,^16^ via alkaline treatment, acidification, and organic phase separation, yielding 60% (w/w) of AAc. Detailed experimental procedures are provided in the Supporting Information.

#### Synthesis of Anacardic Acid-Coated Magnetic Iron Oxide Nanoparticles. Magnetic

Fe_3_O_4_ nanoparticles functionalized with anacardic acid (Fe_3_O_4_@AAc) were prepared by a modified chemical coprecipitation method.^13,15^ Fe­(II) and Fe­(III) salts (2:1 molar ratio) were dissolved in deionized water under nitrogen atmosphere. Anacardic acid dissolved in DMSO was added to the reaction mixture, followed by alkaline precipitation with NH_4_OH at 80 °C. The resulting nanoparticles were magnetically separated, washed with ethanol and water, and dried under vacuum. Detailed experimental conditions are provided in the Supporting Information.

#### Preparation of Nanobiolubricants

Nanobiolubricants were prepared by dispersing Fe_3_O_4_@AAc nanoparticles into trimethylolpropane trioleate (TMPTO, B0) at concentrations of 0.05 wt % (B0.05) and 0.15 wt % (B0.15). The nanoparticles were first redispersed in 30 μL of dichloromethane under ultrasonication (40 kHz, 300 W, 30 min). The suspension was then added to the TMPTO base oil and subjected to further ultrasonication (40 kHz, 300 W, 90 min) to ensure homogeneous dispersion. The solvent was subsequently removed under mild heating at 45 °C for 24 h. Detailed preparation conditions are provided in the Supporting Information.

#### Fourier-Transform Infrared Spectroscopy (FTIR) Analysis

FTIR spectra were recorded using a Bruker Vertex 70v spectrometer. Samples were prepared as KBr pellets (1% w/w sample-to-KBr ratio) and analyzed in the 400–4000 cm^–1^ range, with a resolution of 2 cm^–1^. Each spectrum represents an average of 64 scans, collected at 25 °C under a nitrogen purge to minimize atmospheric interference.

#### Analysis of X-ray Diffraction (XRD)

X-ray diffraction patterns were recorded using a PANalytical X’Pert PRO MPD diffractometer with Co Kα radiation (λ = 1.788 Å), operating at 40 kV and 40 mA. Data were collected in θ–2θ geometry over a 2θ range of 15–80° with a step size of 0.01°. Phase identification was performed using reference diffraction data (ICDD PDF-2), and structural refinement was carried out by the Rietveld method using GSAS-II software.

#### Thermogravimetric Analysis (TGA)

TGA was performed in triplicate using a Shimadzu DTG-60H simultaneous thermal analyzer. Approximately 5 mg of samples were heated from 25 to 700 °C at a rate of 10 °C min^–1^ under nitrogen atmosphere (40 mL min^–1^) using alumina crucibles.

#### Analysis of Scanning Electron Microscope (SEM)

Morphological analysis was conducted using a field-emission gun SEM (Quanta 450, FEI). Samples were ultrasonically dispersed in ethanol, deposited onto silicon substrates, sputter-coated with a thin silver layer, and examined under high vacuum at an accelerating voltage of 10 kV. Particle size distribution was determined from SEM micrographs using ImageJ software.

#### Magnetic Characterization

Magnetic characterization was performed at room temperature using a vibrating sample magnetometer (VSM, Lake Shore, Model 7400, USA), with a maximum applied magnetic field of 12 kOe. The measurement system was calibrated using a pure nickel standard prior to data acquisition. The magnetization was determined after accurate mass measurement and is reported in units of emu/g.

#### Rheological Measurements

Rheological measurements were performed in triplicate using a stress-controlled rheometer (AR 2000, TA Instruments) equipped with a 40 mm cone–plate geometry (1° angle, 47 μm gap). Flow curves were obtained over shear rates from 0 to 1000 s^–1^ at 20, 40, 60, and 80 °C using approximately 0.5 mL of sample. Dynamic viscosity was additionally measured from 10 to 90 °C at a constant shear rate of 10 s^–1^.

#### Tribological Tests

Tribological performance was evaluated using a DHR-3 rheometer (TA Instruments, USA) adapted with a four-ball configuration. Tests were conducted using AISI 52100 chrome steel balls (12.7 mm diameter) under a normal load of 55 N at 75 °C, with a rotational speed of 126 rad s^–1^ (≈1200 rpm) for 60 min. The friction coefficient was continuously recorded, and naphthenic mineral oil (NH-20) was used as a reference lubricant. After testing, wear scar diameter (WSD) was measured, and worn surfaces were examined by optical microscopy (Zeiss, Germany).

#### Evaluation of Nonclinical Safety in Adult Zebrafish (*Danio rerio*)

Nonclinical safety was evaluated in adult zebrafish (*Danio rerio*) of both sexes following oral administration of 20 μL of the pure biolubricant formulations (B0, B0.05, or B15) or untreated control (CN—naive group), according to the methodology described by Collymore, Rasmussen, and Tolwani.^16^ Animals were maintained under controlled conditions and acclimated as previously reported by Magalhães et al.^17^ Locomotor activity was assessed using the open field test, and acute toxicity was determined by monitoring mortality over 96 h postadministration, according to Arellano-Aguilar et al.^18^

All experimental procedures were approved by the Animal Use Ethics Committee of the State University of Ceará (CEUA-UECE, Protocol No. 04009489/2023) and conducted in accordance with CONCEA (2018) guidelines.^19^ Detailed experimental procedures are provided in the Supporting Information.

## Results and Discussion

Fourier-transform infrared spectroscopy (FTIR) was used to confirm the functionalization of Fe_3_O_4_ nanoparticles with anacardic acid (AAc) (Figure S2, Table 1).

**Table 1:** Assignment of the Main FTIR Absorption Bands for Fe_3_O_4_, Anacardic Acid (AAc), and the Fe_3_O_4_@AAc^a^

		Wavenumber (cm^–1^)		
Assignment	Intensity	Fe_3_O_4_	AAc	Fe_3_O_4_@AAc
ν (O–H)	L, M/w	3400–3500	3422	3424
ν (Ar–H)	M	-	3009	-
ν_as_ (C–H)	S	-	2924	2922
ν_s_ (C–H)	M	-	2853	2851
ν (C=O)	S	-	1645	-
δ (C–O)	M	1639	-	-
ν_as_ (COO^–^)	M/S	-	-	1584
ν (C=C)	M	-	1449	1456
ν (C–O)/δ (C–H)/Ar–OH	M	-	1300	-
δ (C–H)	M/S	-	708	-
ν (Fe–O)	S	577	-	577
ν (Fe–O)	M/w	442	-	-

a Abbreviations: ν, stretching; δ, bending; as, asymmetric; s, symmetric; Ar, aromatic; L, large; M, medium; S, strong; w, weak; - , not applicable or not prominently observed.

The spectrum of Fe_3_O_4_ displayed characteristic Fe–O stretching bands at 577 and 442 cm^–1^, corresponding to octahedral and tetrahedral sites of the spinel structure,^20–22^ along with broad absorptions at 3400–3500 cm^–1^ attributed to surface hydroxyl groups and adsorbed water.^22,23^

Pure AAc exhibited the expected vibrational features of its aliphatic and aromatic structure, including C–H stretching bands at 2924 and 2853 cm^–1^ and a carbonyl (C=O) band at 1645 cm^–1^.^24–27^

After functionalization, the Fe–O band at 577 cm^–1^ was preserved, indicating maintenance of the magnetite core. The aliphatic C–H bands remained at 2922 and 2851 cm^–1^, confirming the presence of AAc on the surface. Furthermore, suppression of the C=O band at 1645 cm^–1^, together with the appearance of a band at 1584 cm^–1^ attributed to asymmetric COO^–^ stretching, suggests deprotonation of the carboxylic group and interaction with the Fe_3_O_4_ surface.^26^ The band at 1456 cm^–1^ was assigned to C=C stretching vibrations of the aromatic ring of anacardic acid. These findings confirm successful surface functionalization, consistent with previous reports.^28–30^

X-ray diffraction confirmed the formation of crystalline magnetite (Fe_3_O_4_) with diffraction peaks indexed to the cubic spinel structure (Fd3̅m) corresponding to the (111), (220), (311), (400), (422), (511), (440), and (533) crystallographic planes, in agreement with standard ICSD data (card no. 082433). The characteristic reflections were preserved after functionalization, indicating that anacardic acid coating did not alter the crystalline structure of the nanoparticles^31,32^ (Figure 1A). Rietveld refinement showed good agreement between observed and calculated patterns, supporting the reliability of the structural model. The average crystallite size was estimated as 10.8 ± 1 nm by the Scherrer equation and 13.4 nm using the Williamson–Hall method, with a microstrain of 1.7 × 10^–3^. These values are consistent with those reported by Singh and Goswami^33^ and Ribeiro et al.^13^

**Figure 1:**
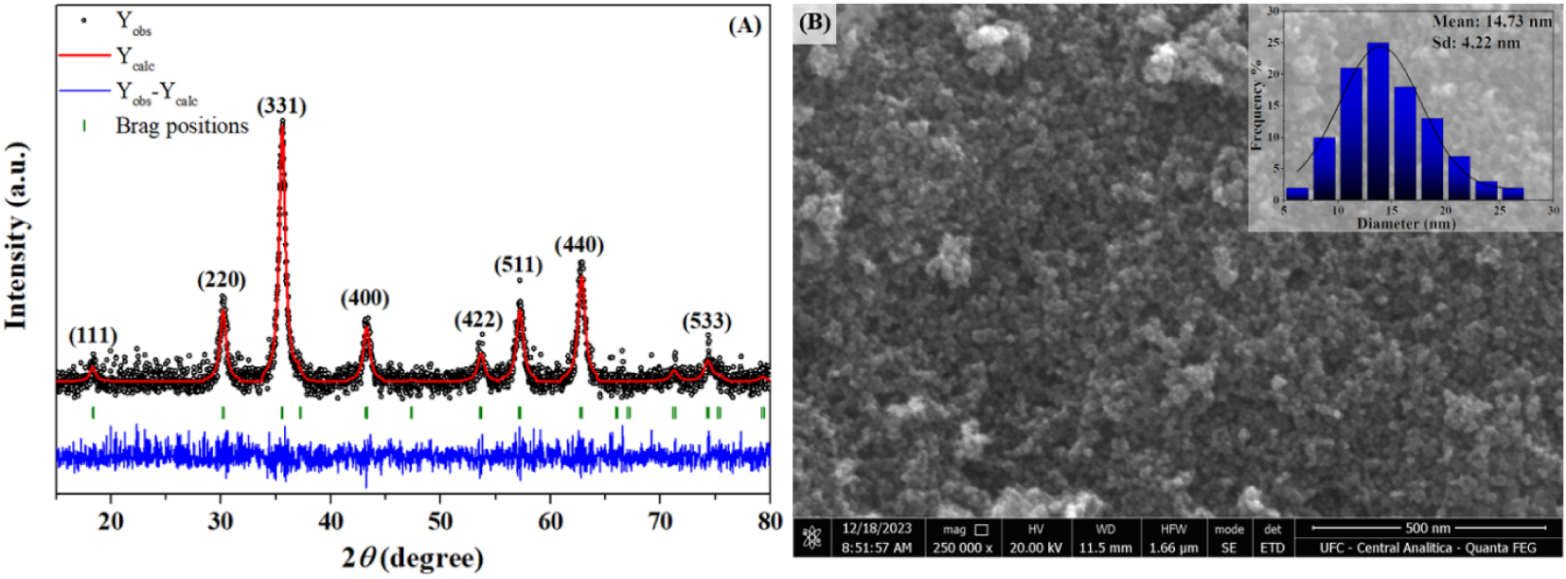
(A) X-ray diffraction (XRD) pattern of Fe_3_O_4_@AAc nanoparticles with Rietveld refinement showing observed (*Y*_obs_), calculated *(Y*_calc_), Bragg peak positions, and difference curve (*Y*_obs_ – *Y*_calc_). (B) SEM micrograph and corresponding particle size distribution histogram of Fe_3_O_4_@AAc nanoparticles.

SEM images (Figure 1B) revealed predominantly spherical nanoparticles with an average diameter of 14.7 ± 4.2 nm (*n* = 100). The slightly larger particle size compared to the crystallite size is attributed to the organic coating and minor aggregation effects. These results are consistent with previous reports for magnetite-based nanomaterials.^13,34^

Regarding the magnetic properties, Figure 2A shows the room temperature magnetization curves for the Fe_3_O_4_ and Fe_3_O_4_@AAc samples. The M­(H) curves exhibit the characteristic reversible S-shaped profile, with negligible coercivity and remanence, features that clearly indicate superparamagnetic behavior and unequivocally confirm this regime for all synthesized samples. This is consistent with the nanometric crystallite size (<20 nm) determined by XRD and SEM analyses, which falls within the typical single-domain regime for magnetite. The magnetization at the maximum applied field for the pure sample is approximately 68.7 emu/g, lower than that of bulk magnetite (*M*_s_ ≈ 84–92 emu/g), as expected from finite-size effects. For the Fe_3_O_4_@AAc nanoparticles, this value decreases to around 32.1 emu/g, reflecting both size-related effects and the nonmagnetic contribution of the anacardic acid coating, which reduces the effective magnetic moment of the composite.

**Figure 2:**
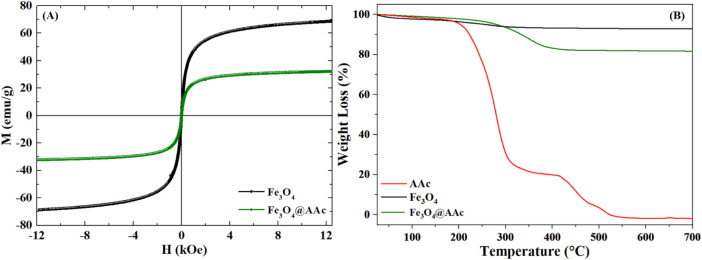
(A) Room-temperature magnetization curves (*M*(*H*)) for the Fe_3_O_4_ (black) and Fe_3_O_4_@AAc (green) samples. (B) TGA curves for AAc (red), uncoated Fe_3_O_4_ (black), and Fe_3_O_4_@AAc (green).

Thermogravimetric analysis was used to evaluate the thermal stability of the base oil (B0) and nanobiolubricants containing Fe_3_O_4_@AAc at 0.05 wt % (B0.05) and 0.15 wt % (B0.15)^35^ (Figure 3A, Table 2). All formulations remained visually homogeneous, without detectable agglomeration or phase separation after synthesis (Figure 3B).

**Figure 3:**
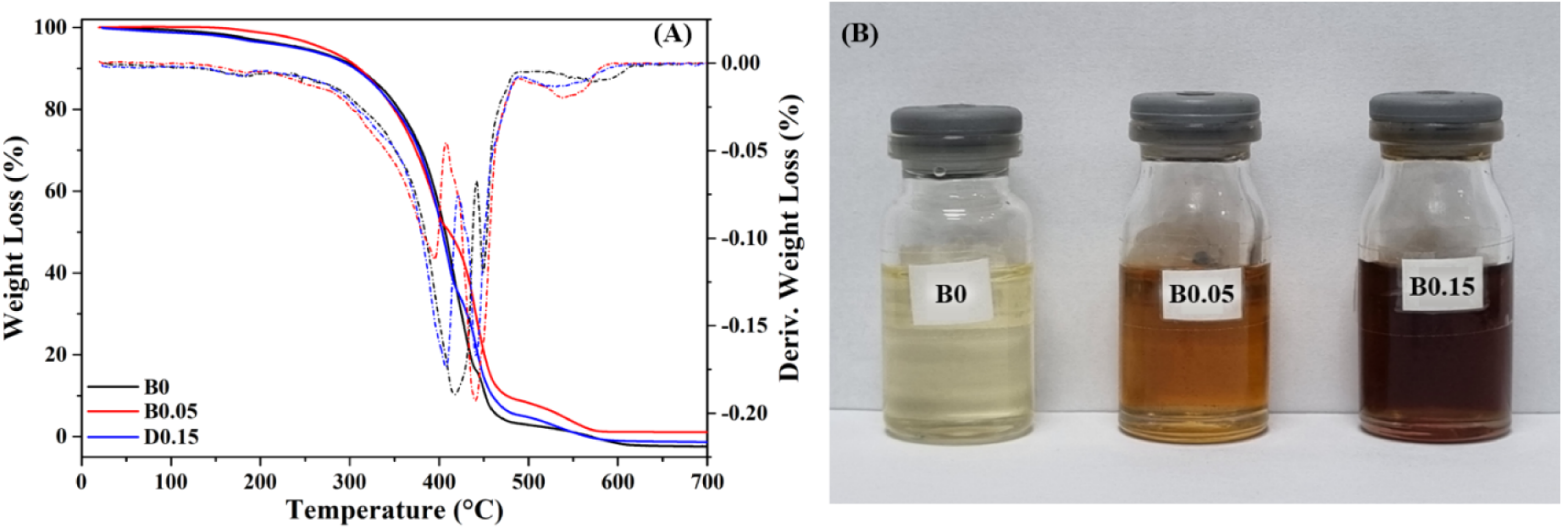
(A) Thermogravimetric analysis (TGA) and derivative thermogravimetric (DTG) curves of biolubricants B0 (black), B0.05 (red), and B0.15 (blue). (B) Photographs of the prepared nanobiolubricant samples: pure TMPTO base oil (B0) and TMPTO containing 0.05 wt % (B0.05) and 0.15 wt % (B0.15) of anacardic acid-functionalized Fe_3_O_4_ nanoparticles (Fe_3_O_4_@AAc).

**Table 2:** Thermogravimetric Data for the Biolubricants

			Peak Temperature (**°**C)		
Samples	*T*_onset_ ^a^ (°C)	Temperature at 50% Mass Loss (**°**C)	1^st^ Event	2^nd^ Event	3^rd^ Event
B0	247.9	406.8	418.2	449.2	574.7
B0.05	270.1	411.6	398.1	440.8	538.1
B0.15	241.8	403.6	409.2	442.4	529.7

a Onset temperature refers to the beginning of thermal degradation during the first decomposition event.

AAc exhibited two main degradation events (∼180 and 420 °C), consistent with previous reports,^13^ while Fe_3_O_4_ showed a mass loss of 6.8% in the 25–700 °C range, attributed to the loss of adsorbed water and surface hydroxyl groups. Fe_3_O_4_@AAc displayed a two-step decomposition profile similar to that of AAc, indicating the presence of the organic coating. After correction for the thermal mass loss of bare Fe_3_O_4_ and assuming complete decomposition of anacardic acid (char yield = 0%), the organic content of the Fe_3_O_4_@AAc nanoparticles was calculated as 12.4 wt %, corresponding to a grafting density of approximately 3.1 molecules/nm^2^. The complete calculation procedure is described in the Supporting Information (Figure 2B).

All biolubricant formulations presented three main degradation stages characteristic of fatty acid esters.^36^ The addition of 0.05 wt % nanoparticles increased the onset degradation temperature (*T*_onset_) from 247.9 °C (B0) to 270.1 °C, indicating enhanced thermal stability.^37^ This improvement is attributed to the antioxidant activity of anacardic acid, whose phenolic hydroxyl groups act as free radical scavengers, interrupting auto-oxidation chain reactions.^2^

In contrast, B0.15 showed a slight reduction in *T*_onset_ (241.8 °C), suggesting a concentration-dependent catalytic effect, as previously observed for metal-based additives.^10^ At higher loadings, the increased surface area and exposed iron sites catalyze biolubricant degradation, while excess nanoparticles may form agglomerates that increase nucleation sites for thermal decomposition.^4^ The highest temperature for 50% mass loss was observed for B0.05, reinforcing the stabilizing effect at moderate nanoparticle loading.

Regarding the apparent inconsistency between *T*_onset_ and *T*_50_, these parameters reflect distinct degradation phenomena. *T*_onset_ is sensitive to the early stages of degradation (where antioxidants act), while *T*_50_ represents the overall decomposition profile, including secondary events and char formation. Thus, the increase in *T*_onset_ for B0.05 confirms antioxidant efficacy, whereas the decrease in *T*_50_ for B0.15 indicates catalytic acceleration after degradation onset.^2,14^

This nonlinear behavior agrees with earlier studies attributing improved stability at low nanoparticle concentrations to barrier and antioxidant effects,^37^ whereas higher loadings may accelerate degradation due to increased nucleation sites.^38^

### Rheology

Rheological properties are crucial in evaluating the biolubricant performance, especially under varying thermal conditions. Viscosity plays a central role, directly influencing lubricating film formation, as well as friction and wear.^14^

Figure 4A shows that dynamic viscosity decreases nonlinearly with temperature rise across all formulations, which is typical of vegetable oil-based lubricants. This thermal response is attributed to the progressive weakening of intermolecular interactions and fluid structure reorganization induced by nanoparticles.^10^

**Figure 4:**
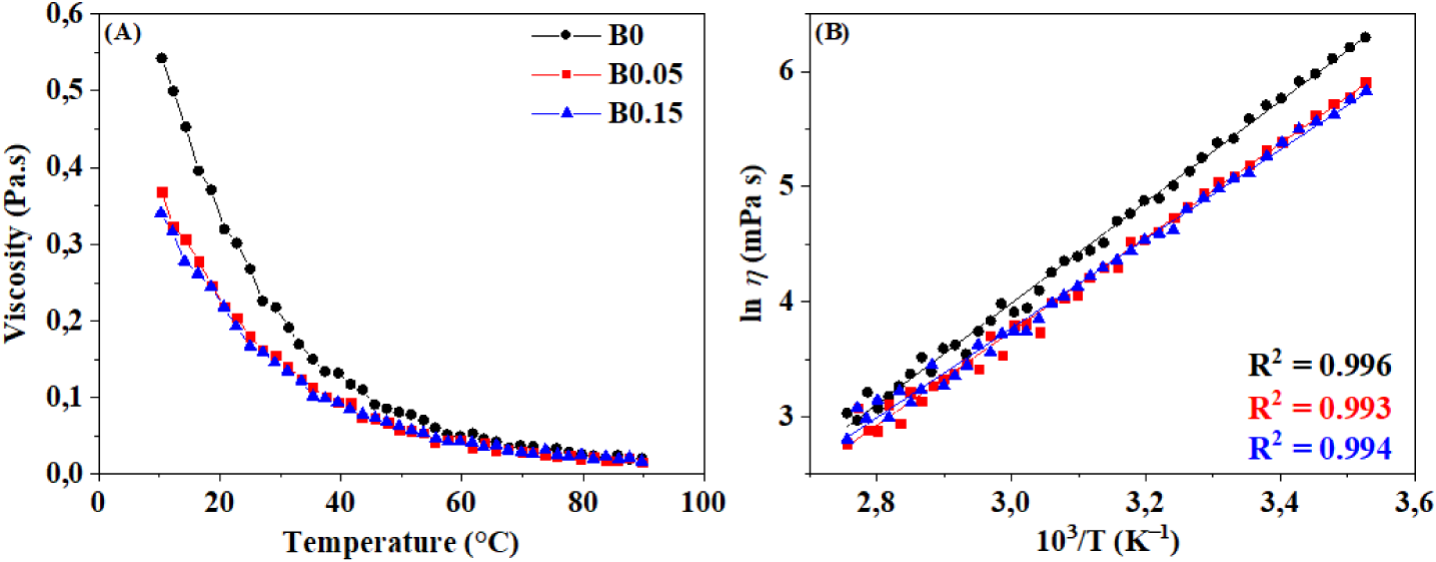
Effect of temperature on the dynamic viscosity behavior of the biolubricants at a constant shear rate of 10 s^–1^. (A) Variation of viscosity as a function of temperature; (B) Arrhenius-type plot (ln η vs 10^3^/*T*) showing the temperature dependence of viscosity and corresponding linear fits (*R* ^2^) for sample B0 (black), B0.05 (red), and B0.15 (blue).

The temperature-dependent viscosity variation was fitted using an Arrhenius equation to calculate the viscous flow activation energy for viscous flow (*E*_a_) (Figure 4B). Lower *E*_a_ values indicate reduced energy barriers for fluid flow at elevated temperatures. The calculated *E*_a_ values were 36.56 kJ/mol (B0), 33.96 kJ/mol (B0.05), and 32.29 kJ/mol (B0.15), showing a gradual decrease with increasing nanoparticle concentration. Nanoparticles act as structuring agents, facilitating molecular movement, as reported by Ahmad et al.^14^ and Wang et al.^10^ for iron oxide nanoparticle systems.

Shear stress versus shear rate behavior and the viscosity responses (see Supporting Information, Figure S3) indicated Newtonian behavior for all biolubricants up to 60 °C. This behavior was evidenced by linear shear stress-shear rate relationships (*R* ^2^ > 0.999) and constant viscosity under shear.

At 80 °C, B0 and B0.05 exhibited non-Newtonian behavior, losing linearity (*R* ^2^ = 0.531 and 0.741, respectively) and showing shear thinning due to lubricant matrix destabilization at high temperatures. In contrast, B0.15 maintained a highly linear response, with an *R* ^2^ = 0.9997 (Table S1), clearly highlighting its excellent agreement with the Newtonian model. This near-unity *R* ^2^ value strongly supports the superior rheological stability and thermal resistance of B0.015 formulation, which may be attributed to improved nanoparticle dispersion and the formation of a more robust microstructure.

Similar behavior was reported by Saka et al.,^39^ who observed that vegetable oils with high oleic acid content exhibit more consistent thermal stability and viscosity. These findings are further aligned with recent reviews, which highlight the influence of optimal nanoparticle concentration on viscosity, emphasizing that inadequate contents can lead to agglomeration and instability.^37^

Tribological performance was evaluated by monitoring the coefficient of friction (COF) over time (Figure 5). The mean COF values were 0.039 ± 0.006 (B0), 0.037 ± 0.008 (B0.05), and 0.036 ± 0.005 (B0.15), comparable to the reference mineral oil NH-20 (0.039 ± 0.002). Among the formulations, B0.15 exhibited more stable friction behavior after ∼1300 s, characterized by smaller COF fluctuations and the onset of steady-state, suggesting the formation and stabilization of a protective tribofilm on the contact surface.

**Figure 5:**
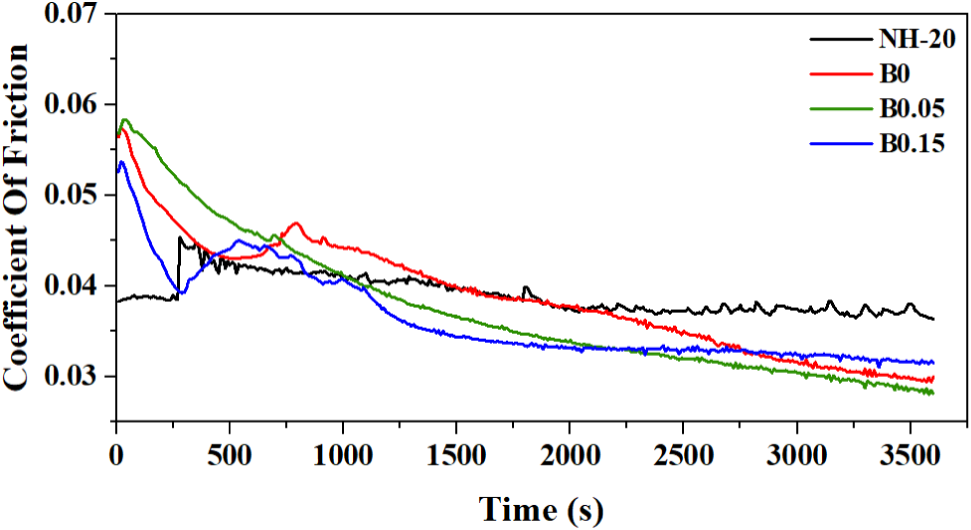
Coefficient of friction versus time for the tested samples.

Normality testing (Shapiro–Wilk, Table S2) indicated non-normal distributions (*p* < 0.001), and the Kruskal–Wallis test confirmed significant differences among groups (*H* = 1440; *p* < 0.001). Pairwise comparisons using the Dwass–Steel–Critchlow–Fligner (DSCF) test (Table S3) showed that nanoparticle-containing formulations significantly reduced COF relative to B0.

The incorporation of Fe_3_O_4_@AAc reduced COF by approximately 8% in B0.15 compared to the base oil. As summarized in Table S4, this reduction is comparable to that reported by Zuin et al.^40^ (∼8%) using 6.7 wt % Fe_3_O_4_ in PAO under similar load conditions, despite the substantially higher nanoparticle concentration. Notably, the same reduction was attained here with only 0.15 wt % Fe_3_O_4_@AAc, representing a 45-fold reduction in additive content. Lineira del Río et al.^41^ reported COF reductions between ∼4% and 18% using 0.015 wt % Fe_3_O_4_ in TMPTO, depending on particle size and coating. Higher reductions (≈30–50%) were observed in mineral and castor-based oils containing 0.5–5 wt % nanoparticles or additional coadditives.^10,14^

These comparisons highlight that anacardic acid functionalization enables efficient dispersion and friction reduction at low nanoparticle loadings, supporting the development of sustainable nanobiolubricants with minimized additive content.

Wear scar diameter (WSD) measurements were conducted using an optical microscope at three magnifications (50×, 100×, and 200×). The average of these measurements was used to define the wear diameter for each sphere (Table 3). The mean WSD values were subjected to the Shapiro–Wilk test, which yielded *p* = 0.969, indicating normal distribution. One-way ANOVA was then performed, revealing a statistically significant difference in wear among the samples (*F*(3, 8) = 13.3; *p* = 0.002), particularly between NH-20 and the other formulations.

**Table 3:**
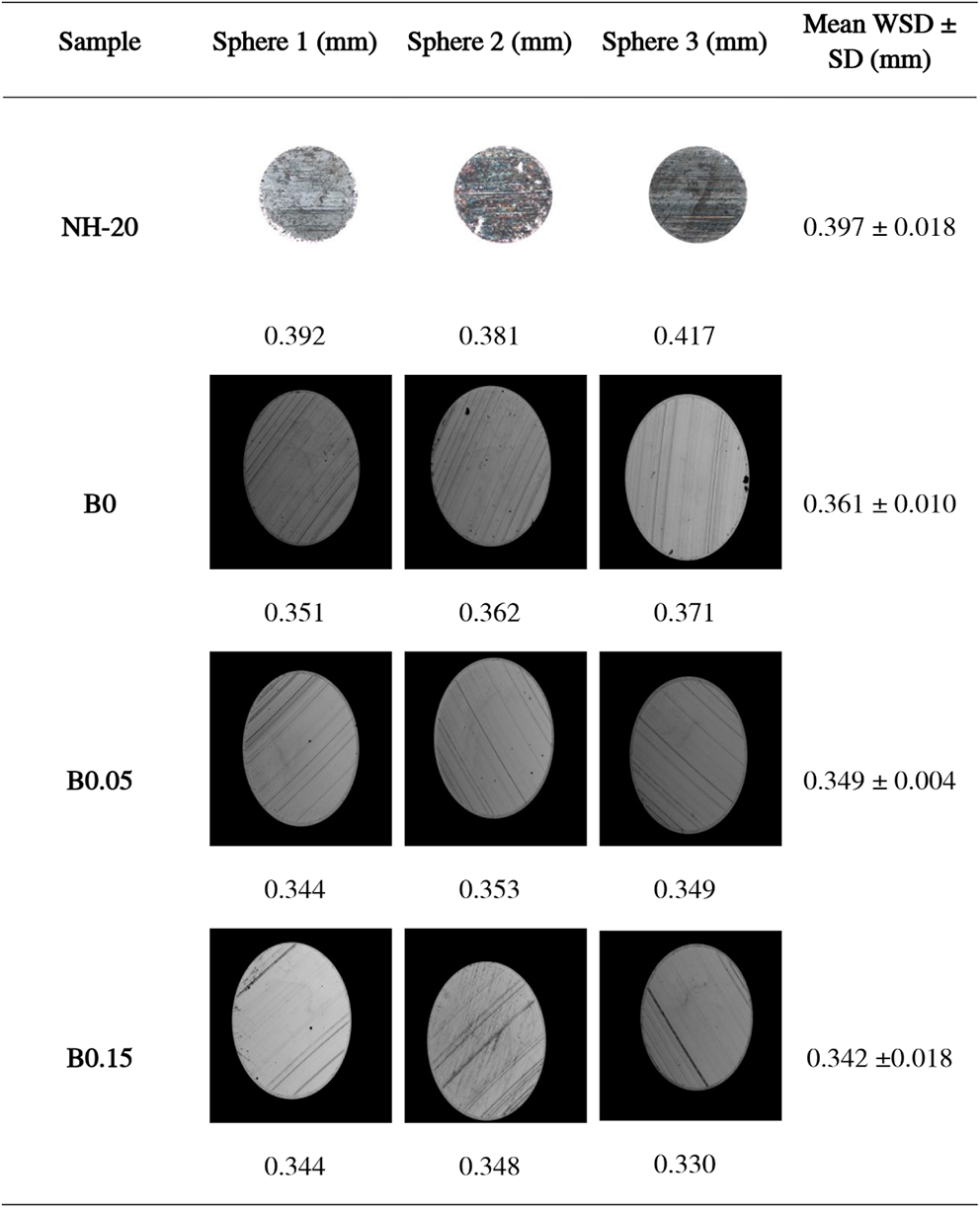
WSD of the Tested Lubricants and Wear Scar Morphology

The Tukey HSD post hoc test for multiple comparisons demonstrated that all biolubricant samples (B0, B0.05, and B0.15) exhibited superior tribological performance compared to NH-20, as evidenced by smaller wear scar diameters. The incorporation of magnetic nanoparticles (in B0.05 and B0.15) resulted in a slight reduction in wear diameter relative to the pure biolubricant (B0). Although these reductions were not statistically significant, the observed trend is consistent with results previously reported by other authors.^10,14^

Although the use of three fixed steel balls for WSD measurements is a valid and widely adopted experimental setup, the lack of statistical significance in WSD reduction may be addressed in future studies by increasing the number of replicates to enhance statistical power. Additionally, the use of laser profilometry is suggested for higher precision in wear measurements, as it provides more accurate topography data compared to optical microscopy.

This trend in reduction can be explained by different interaction mechanisms reported in literature. Iron oxide nanoparticles promote the formation of a protective tribofilm, reducing direct contact between surface asperities and, consequently, material loss. Guo et al.^5^ observed that this process occurs through the mechanism of tribosintering, in which the nanoparticles, under shear stress, aggregate to form a dense and cohesive film of Fe_3_O_4_ nanoparticles that acts as a protective physical barrier. They may also act as rolling elements (“ball-bearing effect”), decreasing shear forces at the contact interface.^10^

The B0.15 formulation simultaneously presented a slight reduction in friction and greater wear resistance, demonstrating the synergistic effect of incorporating Fe_3_O_4_@AAc nanoparticles and reinforcing their potential to optimize the tribological performance of TMPTO.

The locomotor activity assay showed that zebrafish swimming performance remained above 67% of the naïve control group, with no significant differences among treatments (*p* > 0.05; Figure 6). These findings indicate that neither the pure biolubricant (B0) nor the Fe_3_O_4_@AAc-containing formulations induced acute neurobehavioral alterations, supporting the biosafety of the developed systems under the tested conditions.

**Figure 6:**
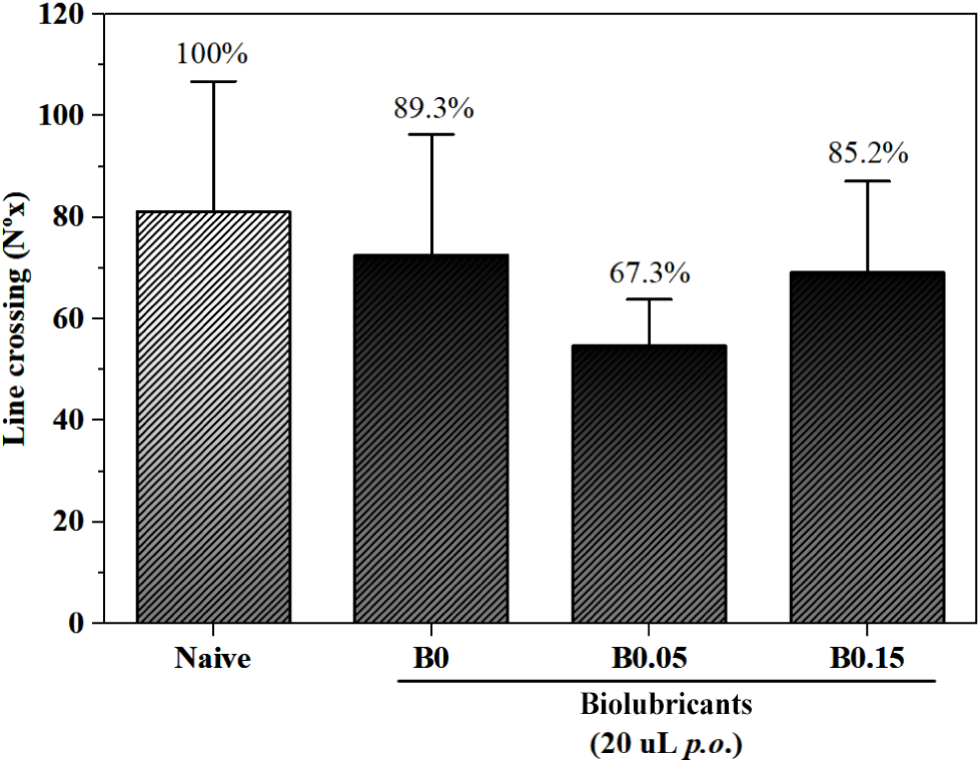
Evaluation of the neurobehavioral potential of biolubricants in adult zebrafish. Locomotor activity, quantified by the number of line crossings (CL) in a 5 min open field test, was analyzed after oral administration of the biolubricants. None of the formulations, pure biolubricant (B0) or supplemented with nanoparticles (B0.05 and B0.15), induced significant changes in locomotor activity when compared to the untreated control group (Naive) (*p* > 0.05). Data are expressed as mean ± SEM (*n* = 6 animals/group). Numbers above the bars indicate the percentage of locomotor activity. Statistical analysis was performed using one-way ANOVA followed by Tukey’s post hoc test, with specific comparisons to the Naive group (* *p* < 0.05).

In the 96 h acute toxicity test, no mortality was observed in the nanoparticle-containing groups, while only one isolated case occurred in B0 (see Supporting Information, Table S5), indicating that nanoparticle incorporation did not increase the toxicity of TMPTO.

Unlike previous reports describing neurobehavioral alterations after exposure to uncoated iron oxide nanoparticles or parenteral administration routes,^24,42–45^ no adverse behavioral or acute toxicological effects were detected in the present study. This improved safety profile is likely associated with the surface functionalization of Fe_3_O_4_ nanoparticles with anacardic acid, which may reduce nanoparticle surface reactivity and biological interactions. In addition, the biolipid matrix of the TMPTO-based formulation and the oral administration route may further contribute to lowering nanoparticle bioavailability and toxicity.^46^ These findings suggest a potential safety advantage of surface-functionalized Fe_3_O_4_ nanoparticles compared with uncoated systems.

## Conclusions

The synthesis of magnetic iron oxide nanoparticles coated with anacardic acid (Fe_3_O_4_@AAc) was successfully achieved, with coating confirmation by FTIR and characterization of the crystalline structure and crystallite size by XRD. The spherical and homogeneous morphology of the nanoparticles was evidenced by SEM, which had an average size of 14.7 nm ± 4.2 nm. Thermal analysis indicated enhanced stability of the coated nanoparticles, with approximately 12.4 wt % anacardic acid present on the surface. In the biolubricants, adding 0.05 wt % of the nanoparticles (B0.05) increased thermal resistance, whereas 0.15 wt % (B0.15) had a catalytic effect, lowering the degradation onset temperature to values close to those of the biolubricant without nanoparticles.

From a rheological perspective, all formulations exhibited the expected vegetable oil behavior, with viscosity decreasing as temperature increased. The nanoparticle addition reduced the activation energy for viscous flow, particularly at higher concentrations (0.15%), and provided greater rheological stability at elevated temperatures by maintaining Newtonian behavior up to 80 °C.

Tribological results demonstrated that the formulation containing 0.15 wt % nanoparticles (B0.15) had the lowest coefficient of friction (COF = 0.036 ± 0.005) and formed a more stable and enduring tribofilm. This formulation outperformed both the neat biolubricant and the reference mineral oil (NH-20). Although no statistically significant differences were observed, all formulations containing nanoparticles showed a reduction in wear scar diameter, demonstrating superior performance compared to NH-20 and indicating the effectiveness of the nanoparticles in mitigating friction and wear.

The formulations B0, B0.05, and B0.15 showed no neurobehavioral effects or acute toxicity in adult zebrafish, indicating a low immediate risk of toxicity. These results suggest that the lipid matrix of the biolubricant may reduce potential adverse effects of the nanoparticles, providing the formulations with a safer profile for future applications.

In summary, the addition of Fe_3_O_4_@AAc nanoparticles significantly enhanced the tribological properties of the biolubricant by reducing friction, improving the stability of the lubricating film, and decreasing surface wear. This demonstrates their potential as a sustainable functional additive for biolubricant formulations.

## Supplementary Material



## Notes

Experimental procedures for CNSL extraction, anacardic acid isolation, synthesis of Fe_3_O_4_@AAc nanoparticles, preparation of nanobiolubricants, zebrafish biosafety assays, crystallite size and microstrain calculations from XRD data, and determination of anacardic acid content and grafting density from TGA data; Figure S1: chemical structures of trimethylolpropane trioleate and anacardic acid mixture; Figure S2: FTIR spectra of Fe_3_O_4_ anacardic acid, and Fe_3_O_4_ @AAc nanoparticles; Figure S3: shear stress versus shear rate and viscosity behavior of B0, B0.05, and B0.15 formulations; Table S1: linear regression parameters of flow curves; Table S2: descriptive statistics and normality analysis of coefficient of friction measurements; Table S3: pairwise comparisons using the Dwass–Steel–Critchlow–Fligner test; Table S4: comparison of coefficient of friction reduction reported for nanoparticle-based lubricants; Table S5: acute toxicity evaluation in adult zebrafish after 96 h exposure (PDF)
