# Supplementary material for: Anacardic Acid-Modified Fe3O4 Nanoparticles: Synthesis, Characterization, and Thermal and Tribological Performance in Biolubricants
Source: ACS Omega. 2026 Jul 8;11(28):42484–92. doi: 10.1021/acsomega.6c03439 (PMC13393022; doi:10.1021/acsomega.6c03439)
Supplement: Supplementary file 1 [file ao6c03439_si_001.pdf]

Anacardic Acid-Modified Fe_3O_4 Nanoparticles: Synthesis, Characterization, and Thermal and Tribological Performance in Biolubricants

David Alves de Assis[a], *Denise Ramos Moreira**[a], *Alexandre Carreira da Cruz Sousa*[ab], *Antonia Flávia Justino Uchoa*[c], *Felipe Bohn*[d], *Walney Silva Araujo*[e], *and Nágila Maria Pontes Silva Ricardo**[a]

[a] Laboratory of Polymers and Materials Innovation, Department of Organic and Inorganic Chemistry, Federal University of Ceará, 60440-900, Fortaleza, Ceará, Brazil

[b] Federal Institute of Education, Science and Technology of Ceará, Quixadá Campus, 63902-580 Quixadá, Ceará, Brazil

[c] Department of Transportation, Federal University of Ceará, 60020-181 Fortaleza, Ceará, Brazil

[d] Department of Physics, Federal University of Rio Grande do Norte, 59078-900 Natal, Rio Grande do Norte, Brazil

[e] Department of Metallurgical and Materials Engineering, Federal University of Ceará, 60440-554 Fortaleza, Ceará, Brazil

*Corresponding author: Nágila M. P. S. Ricardo - Laboratory of Polymers and Materials Innovation, Federal University of Ceara, Fortaleza 60455-760, Brazil; Tel: +55 85 3366 9142; e-mail: naricard@ufc.br.

Denise R. Moreira - Laboratory of Polymers and Materials Innovation, Federal University of Ceara, Fortaleza 60455-760, Brazil; Tel: +55 85 3366 9142; e-mail: denisemoreira@alu.ufc.br.

Number of pages: 11
Number of Figures: 3
Number of Tables: 5

Extraction of CNSL and Isolation of Anacardic Acid

Extraction of Cashew Nut Shell Liquid (CNSL)

CNSL was extracted according to Philip et al.[15] with modifications. Cashew nuts were washed, oven-dried at 45 °C, and manually cracked to remove the kernels. The shells were peeled and ground into fine particles. A total of 76.8 g of shells were immersed in 200 mL of ethyl acetate and macerated for 72 h at room temperature (25 ± 2 °C).

The mixture was filtered to remove solid residues, and the filtrate was concentrated under reduced pressure at 75 °C to remove the solvent. CNSL was obtained as a viscous liquid (15.6 g, 20.3% w/w yield).

Isolation of Anacardic Acid (AAc)

AAc was isolated following Paramashivappa et al.[16] with minor modifications. CNSL (10 g) was dissolved in 50 mL of 95% (v/v) ethanol containing $Ca(OH)_2$ (5 g) and stirred at 50 °C for 3 h. The resulting precipitate was collected by vacuum filtration and washed with ethanol to remove unreacted CNSL.

The solid was transferred to a separatory funnel and treated with 50 mL of 10% (v/v) HCl, followed by 50 mL of ethyl acetate. After vigorous shaking, the organic phase was separated, dried over anhydrous Na_2SO_4, and concentrated under reduced pressure.

The final yield of the AAc mixture was 6 g (60% w/w).

Synthesis of Anacardic Acid-Coated Magnetic Iron Oxide Nanoparticles (Fe_3O_4@AAc)

Magnetic iron oxide nanoparticles functionalized with anacardic acid (Fe_3O_4@AAc) were synthesized by chemical co-precipitation following previously reported procedures[13],[15] with modifications.

Briefly, 0.8 g of iron(II) chloride tetrahydrate ($FeCl_2 \cdot 4H_2O$, 4,0 mmol) and 2.2 g of iron(III) chloride hexahydrate ($FeCl_3 \cdot 6H_2O$, 8,1 mmol) were dissolved in 100 mL of deionized water in a three-neck round-bottom flask under continuous nitrogen flow. The mixture was mechanically stirred at 500 rpm and maintained at 50 °C for 10 min to ensure complete dissolution. The Fe^{2+}/Fe^{3+} molar ratio was verified as 1:2, consistent with the stoichiometric ratio required for magnetite formation.

Anacardic acid (2 g) was dissolved in 10 mL of dimethyl sulfoxide (DMSO) and added dropwise to the iron salt solution under nitrogen atmosphere. The reaction temperature was increased to 80 °C, and 10 mL of 28% (w/w) ammonium hydroxide (NH_4OH) was rapidly injected under vigorous stirring (1000 rpm) to induce precipitation. The reaction was maintained at 80 °C for 1 h.

The resulting black precipitate was magnetically separated and washed sequentially with 96% ethanol (3 × 50 mL) and Milli-Q water until neutral pH (≈7) was achieved. The nanoparticles were dried under vacuum and stored in a desiccator until further use.

Preparation of Nanobiolubricants

Nanobiolubricant formulations were prepared by incorporating Fe_3O_4@AAc nanoparticles into trimethylolpropane trioleate (OTMPE), designated as B0. For each formulation, a total mass of 30 g was prepared, targeting nanoparticle concentrations of 0.05 wt% (B0.05) and 0.15 wt% (B0.15).

The required mass of nanoparticles was accurately weighed using a Shimadzu AUW220D analytical balance (± 0.01 mg). The nanoparticles, which had been previously dried and stored in a desiccator, were redispersed in 30 µL of dichloromethane (CH_2Cl_2) using an ultrasonic bath operating at 40 kHz and 300 W for 30 min to promote complete disagglomeration.

The resulting suspension was added to the OTMPE base oil and subjected to further ultrasonication (40 kHz, 300 W) for 90 min to ensure homogeneous dispersion. The formulations were then placed in a forced-air circulation oven at 45 °C for 24 h to ensure complete removal of dichloromethane.

Stable nanoparticle dispersions were obtained without visible agglomeration or residual solvent.

Non-Clinical Safety Evaluation in Adult Zebrafish (*Danio rerio*)

Animals and Husbandry Conditions

Adult zebrafish (Danio rerio) of both sexes (60–90 days old; body length 3.5 ± 0.5 cm; body mass 0.4 ± 0.1 g) were used in the experiments. Upon arrival, the animals were acclimated for 24 h in rectangular glass aquaria (30 × 15 × 20 cm) according to the protocol described by Magalhães et al.[15]

Tanks contained previously dechlorinated water (ProtecPlus®), continuously aerated using air pumps and submerged filters, maintained at 25 °C and neutral pH. Fish were kept under a 14/10 h light/dark cycle and fed ad libitum until 24 h prior to the assays.

Sample Administration

At the beginning of the experiment, animals were randomly selected and immobilized using moistened sponge holders. Oral administration (p.o.) of 20 µL of the test sample was performed according to the methodology described by Collymore, Rasmussen, and Tolwani.[16]

The following groups were included:

(i) untreated (naïve) group;

(ii) pure trimethylolpropane trioleate biolubricant (B0);

(iii) biolubricant with 0.05 wt% Fe_3O_4@AAc (B0.05);

(iv) biolubricant with 0.15 wt% Fe_3O_4@AAc (B0.15).

No solvents were used when preparing the samples for the biosafety assays.

Locomotor Activity Test (Open Field)

Specimens (n = 6/group) received 20 μL of the pure biolubricant formulations (B0, B0.05, or B0.15) by oral administration and were individually maintained for 1 h to allow absorption of the administered solution.

The locomotor activity assay was conducted in Petri dishes (10 × 15 cm) divided into four quadrants and filled with system water. Fish were individually transferred to the apparatus and observed for 5 min. Locomotor activity was quantified by recording the number of line crossings (LC) between quadrants, following a methodology adapted from Magalhães et al.[15]

Acute Toxicity (96 h)

For the acute toxicity assay, specimens (n = 6/group) were treated orally with 20 μL of the pure biolubricant formulations (B0, B0.05, or B0.15) or an untreated control (CN – naive group). After treatment, animals were monitored for 96 h to record mortality. As the samples were administered in pure form without dilution, no LC_{50} calculation was performed, according to the method described by Arellano-Aguilar et al.[18]

Euthanasia and Ethical Approval

At the end of the experimental procedures, fish were euthanized by immersion in ice-cold water (2–4 °C) for 10 min, until complete cessation of opercular movements, in strict accordance with CONCEA (2018) guidelines.[17]

All experimental procedures were previously approved by the Animal Use Ethics Committee of the State University of Ceará (CEUA-UECE), under protocol No. 04009489/2023.

Crystallite Size and Microstrain Analysis from XRD Data

The crystallite size of Fe_3O_4@AAc nanoparticles was estimated using the Scherrer equation (Eq. S1):

$$D_S = \frac{K\,\lambda}{\beta coscos\,\theta} \qquad \text{(Eq 1)}$$

Where D_S is the crystallite size calculated using the Scherrer equation, K is a constant dependent on reflection symmetry (K = 0.9), $\lambda$is the X-ray wavelength (λ = 0,15406 nm), β is the full width at half maximum (FWHM) of the diffraction peak, and θ is the Bragg angle.

Microstrain and crystallite size were also determined using the Williamson–Hall (W–H) method (Eq. S2):

$$\beta\ cos\theta = \frac{K\,\lambda}{D_{W-H}} + \frac{4\,\varepsilon\ sen\theta}{\lambda} \qquad \text{(Eq 2)}$$

Where D_{W-H} is the crystallite size obtained from the W–H method and ε represents the microstrain.

Calculation of nnacardic acid content and grafting density on Fe_3O_4@AAc nanoparticles

Thermogravimetric analysis (TGA) data

Thermogravimetric analysis was performed from 25 °C to 700 °C under nitrogen atmosphere. Pure anacardic acid (AAc) showed complete mass loss during heating, with an initial mass of 4.71045 mg and no residual mass at 700 °C. Pure Fe_3O_4 presented an initial mass of 9.43053 mg and a final mass of 8.78875 mg, corresponding to a mass loss of 0.64178 mg (6.81%). For the functionalized nanoparticles (Fe_3O_4@AAc), the initial and final masses were 10.26226 mg and 8.37648 mg, respectively, resulting in a total mass loss of 1.88578 mg.

Calculation of anacardic acid content

- The calculation of the anacardic acid content on the functionalized nanoparticles was performed considering the following assumptions:
- Pure AAc completely decomposes up to 700 °C, i.e., char yield = 0%;
- Pure Fe_3O_4 exhibits a mass loss of 6.81% in the 25–700 °C range, attributed to the loss of adsorbed water and surface hydroxyl groups;
- The final mass of the Fe_3O_4@AAc sample at 700 °C corresponds exclusively to the iron oxide core.

Step 1: Initial mass of Fe_3O_4 in the Fe_3O_4@AAc sample

Since pure Fe_3O_4 loses 6.81% of its mass upon heating, the final mass (8.37648 mg) corresponds to 93.19% of the initial Fe_3O_4 mass:

$$Initial\ Fe3O4\ mass = \ 8.37648/0.9319 = 8.989\ mg$$

Step 2: Mass of AAc in the Fe_3O_4@AAc sample

$$AAc\ mass = \ Initial\ Fe3O4@AAc\ mass - Initial\ Fe3O4\ mass$$

$$AAc\ mass = \ 10.26226\ mg - 8.989\ mg = 1.273\ mg$$

Step 3: Weight percentage of AAc

$$AAc\ content\ (\%) = ((1.273\ mg)/(10.26226\ mg)) \times 100\% = 12.4\%$$

Thus, the functionalized nanoparticles contain 12.4 wt% of anacardic acid.

Calculation of grafting density (molecules/nm²)

The grafting density of anacardic acid (AAc) on the Fe_3O_4 nanoparticles was estimated using the average nanoparticle diameter obtained by TEM (14.7 nm), the density of Fe_3O_4 reported in the literature (5.12 g/cm³), the average molar mass of AAc determined by HPLC analysis (345 g/mol), and the AAc content obtained from TGA (12.4 wt%). Avogadro's constant (6.022×10^{23} mol⁻¹) was used for the conversion from moles to molecules.

Step 1: Specific surface area of the Fe_3O_4 core

For spherical monodisperse particles, the specific surface area (S) per gram of Fe_3O_4 is:

$S=6/(\rho\times d)$

$$S = 6/(5.12\ g/cm^3 \times 1.47 \times 10^{-6}\ cm) = 7.97 \times 10^{5}\ cm^2/g$$

$$S = 7.97 \times 10^{19}\ nm^2/g$$

Step 2: Mass of Fe_3O_4 in 1 g of Fe_3O_4@AAc sample

$$Fe3O4\ mass = 1\ g \times (1 - 0.124) = 0.876\ g$$

Step 3: Total surface area of the Fe_3O_4 core in 1 g of sample

$$Total\ area = Fe3O4\ mass \times S$$

$$Total\ area = 0.876\ g \times (7.97 \times 10^{19}\ nm^2/g) = 6.98 \times 10^{19}\ nm^2$$

Step 4: Number of AAc molecules in 1 g of sample`

$$n_{AAc} = m_{AAc}/(Average\ molar\ mass\ of\ AAc)$$

$$n_{AAc} = (0.124\ g)/(345\ g\ mol^{-1})$$

$$n_{AAc} = 3.594 \times 10^{-4}\ mol$$

Total number of AAc molecules:

$$T_{Moles} = n_{AAc} \times N_a$$

$$T_{Moles} = 3.594 \times 10^{-4}\ mol \times 6.022 \times 10^{23}\ mol^{-1}$$

$$T_{Moles} = 2.16 \times 10^{20}\ molécules$$

Step 5: Grafting density (δ)

$$\delta = n_{AAc}/(Total\ area)$$

$$\delta = (2.16 \times 10^{20}\ molécules)/(6.98 \times 10^{19}\ nm^2)$$

$$\delta = 3.07\ (molécules)/(nm^2)$$

Result: The grafting density of anacardic acid on the Fe_3O_4 nanoparticles is approximately 3.1 molecules/nm².

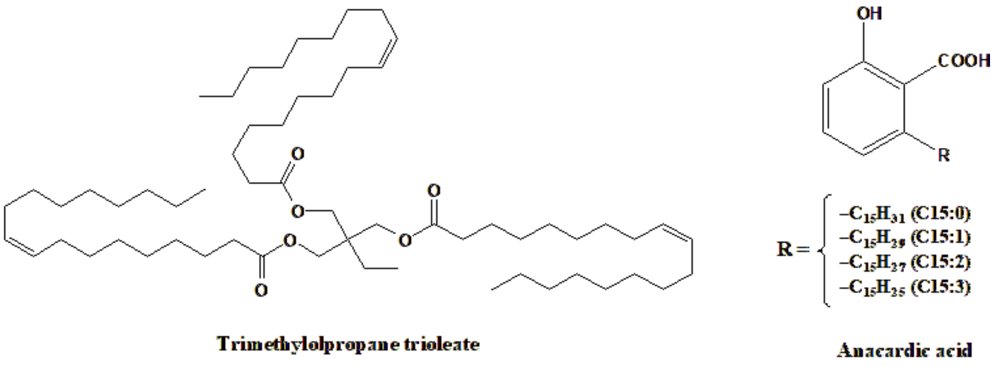

Figure S1. Chemical structure of trimethylolpropane trioleate (left) and anacardic acid mixture (right).

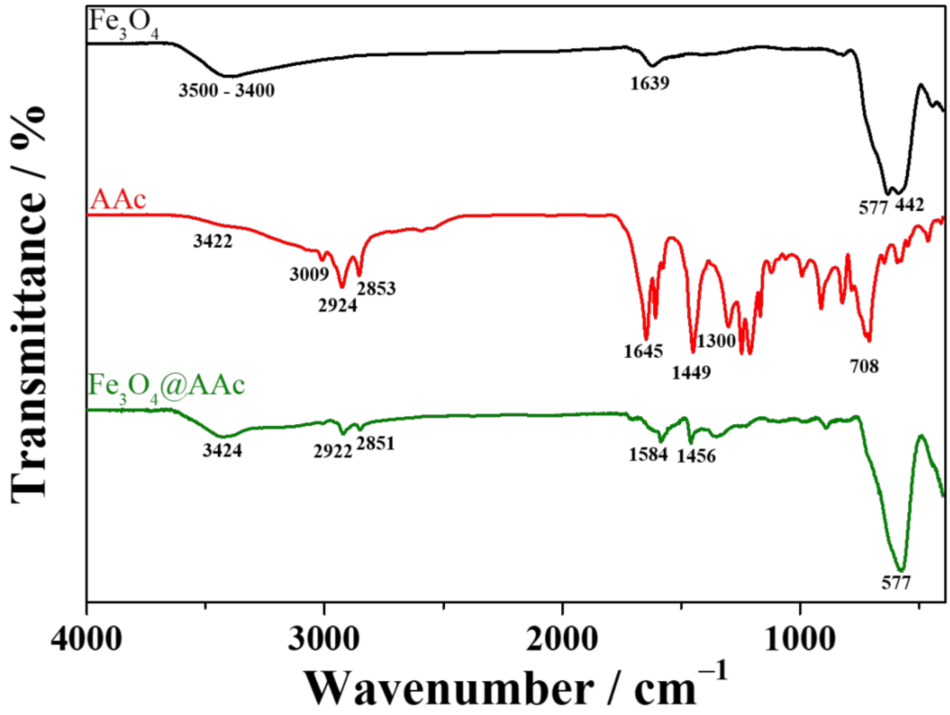

Figure S2. FTIR spectra of Fe_3O_4 nanoparticles, anacardic acid (AAc), and Fe_3O_4@AAc nanoparticles.

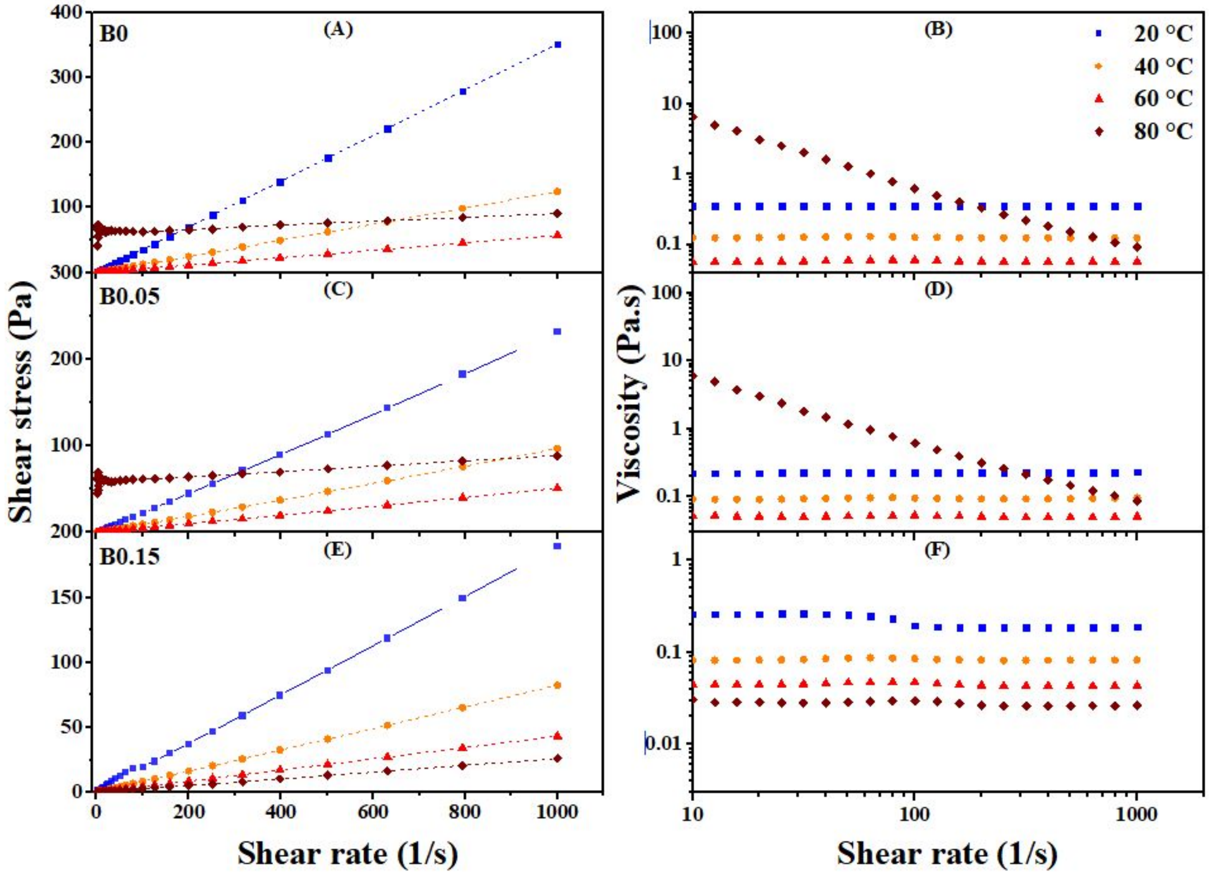

Figure S3. Shear stress vs. shear rate and the effect of shear rate on viscosity behavior for B0 (A and B), B0.05 (C and D), and B0.15 (E and F)

Table S1 - Linear regression analysis of the flow curves for B0, B0.05, and B0.15 samples

Sample: B0					
**Temperature (°C)**	**Adjusted R^2**	**Intercept (a)**	**Std. Error (a)**	**Slope (b)**	**Std. Error (b)**
20	1.00000	0.15175	0.03182	0.35161	1.07638×10^{-4}
40	0.99999	0.06474	0.02558	0.12410	8.65109×10^{-5}
60	0.99995	0.03932	0.02081	0.05712	7.03881×10^{-5}
80	0.53103	61.16396	1.46664	0.02934	4.96000×10^{-3}
**Sample: B0.05**					
**Temperature (°C)**	**Adjusted R^2**	**Intercept (a)**	**Std. Error (a)**	**Slope (b)**	**Std. Error (b)**
20	0.99985	-0.29179	0.15185	0.23010	5.13609×10^{-4}
40	0.99976	-0.13315	0.07922	0.09573	2.67959×10^{-4}
60	0.99983	0.01264	0.03610	0.05070	1.22113×10^{-4}
80	0.74080	58.93356	0.92544	0.02915	3.13000×10^{-3}
**Sample: B0.15**					
**Temperature (°C)**	**Adjusted R^2**	**Intercept (a)**	**Std. Error (a)**	**Slope (b)**	**Std. Error (b)**
20	0.99947	1.20660	0.23163	0.18655	7.83456×10^{-4}
40	0.99996	0.04282	0.02829	0.08205	9.56888×10^{-5}
60	0.99989	0.08110	0.02447	0.04310	8.27506×10^{-5}
80	0.99970	0.07237	0.02425	0.02602	8.20100×10^{-5}

Table S2 - Mean values, Shapiro-Wilk test (p-values), median, first quartile (25%), and third quartile (75%) for COF measurements of the samples.

Sample	Mean ± SD	Shapiro-Wilk (p)	Median	1st Quartile (25%)	3rd Quartile

**NH-20**	0.039 ± 0.002	<0.001	0.0385	0.0373	0.0407
**B0**	0.039 ± 0.006	<0.001	0.0385	0.0334	0.0436
**B0.05**	0.038 ± 0.008	<0.001	0.0347	0.0314	0.0423
**B0.15**	0.036 ± 0.005	<0.001	0.0336	0.0328	0.0404

Table S3. Pairwise comparisons using the Dwass–Steel–Critchlow–Fligner (DSCF) test (p-values) among the samples

**Comparison**	**DSCF (p)**	**Comparison**	**DSCF (p)**
NH-20 vs B0	0.778	B0 vs B0.05	<0.001
NH-20 vs B0.05	<0.001	B0 vs B0.15%	<0.001
NH-20 vs B0.15	<0.001	B0.05 vs B0.15%	0.954

Table S4: Comparison of COF reduction in different studies with nanoparticle-based lubricants.

**Study**	**Lubricant base**	**NP concentration**	**COF reduction**
This work	Trimethylolpropane triester with oleic acid (OTMPE)	0.05% - 0.15% Fe_3O_4@AAc	≈ 8%
Zuin et al. (2017)	Polyalphaolefin (PAO8)	6.7% Fe_3O_4 (coated with stearic acid)	≈ 8%
Liñeira del Río et al. (2020)	Trimethylolpropane trioleate (TMPTO)	0.015% Fe_3O_4 (6.3 nm - 10 nm)	≈ 4% and 18%
Wang et al. (2023)	Mineral oil	5% Fe_3O_4 (oleylamine)	≈ 30%
Ahmad et al. (2022)	Modified Castor Seed Oil (MCSO)	0.5% Fe_3O_4<br>0.5% Fe_3O_4 + ethyleneglycol	≈ 33% and 50%

Legend: Comparison of the effect of iron oxide nanoparticles (Fe_3O_4) on the coefficient of friction (COF) across different lubricant bases. Approximate values as reported in the literature. Source: Adapted from Zuin et al. (2017), Liñeira del Río et al. (2020), Wang et al. (2023), Ahmad et al. (2022), and results of this work.

Table 5. Determination of 96 h Toxicological Activity of the Biolubricant (B0) with Different Concentrations of Magnetic Nanoparticles

Samples	Adult Zebrafish Mortality after 96 h	
	CN	C1
**B0**	0	1
**B0.05**	0	0
**B0.15**	0	0

Legend: B0 – Trimethylolpropane oleate biolubricant; B0.05 – Trimethylolpropane oleate biolubricant supplemented with 0.05% (w/w) magnetic nanoparticles; DB15 – Trimethylolpropane oleate biolubricant supplemented with 0.15% (w/w) magnetic nanoparticles; CN – Negative control group: distilled water; C1 – Concentration group (20 μL of the pure sample).
